# Characterization of the Four *Rosa* L. Species from Kazakhstan Based on Complete Plastomes and Nuclear Ribosomal Internal Transcribed Spacer (*ITS*) Sequences

**DOI:** 10.3390/genes16080852

**Published:** 2025-07-22

**Authors:** Moldir Yermagambetova, Akzhunis Imanbayeva, Margarita Ishmuratova, Aidar Sumbembayev, Shyryn Almerekova

**Affiliations:** 1 Institute of Plant Biology and Biotechnology, Almaty 050040, Kazakhstan; ermaganbetova.moldir@bk.ru; 2 Mangyshlak Experimental Botanical Garden, Aktau 130000, Kazakhstan; imangarden@mail.ru; 3 Biology and Geography Faculty, Karaganda Buketov University, Karaganda 100028, Kazakhstan; margarita.ishmur@mail.ru; 4 Altai Botanical Garden, Ridder 071300, Kazakhstan; aydars@list.ru

**Keywords:** comparative analysis, *ITS*, Kazakhstan, phylogeny, plastome, *Rosa*, *ycf1*

## Abstract

Background: *Rosa* L. is an economically significant genus with species that are notable for their rich content of phenolic compounds. Despite its importance, the taxonomy of *Rosa* remains complex and unresolved. Methods: We sequenced, assembled, and performed comparative analyses of the complete plastomes of four *Rosa* species: *R. acicularis*, *R. iliensis*, *R. laxa*, and *R. spinosissima*. In addition to the plastome, we sequenced the nuclear ribosomal internal transcribed spacer (*ITS*). Results: Plastomes ranged in size from 157,148 bp (*R. iliensis*) to 157,346 bp (*R. laxa*). In each plastome, 136 genes were annotated, comprising 90 protein-coding, 38 tRNA, and eight rRNA genes. A total of 905 SSRs were identified, ranging from 224 (*R. acicularis*) to 229 in *R. spinosissima*. Nine highly variable regions were detected, including two coding genes (*rps16* and *ycf1*) and seven intergenic spacers (*ycf3-trnS*(*GGA*), *trnT*(*UGU*)*-trnL*(*UAA*), *rpl14-rpl16*, *trnR*(*UCU*)*-atpA*, *trnD*(*GUC*), *trnG*(*UCC*)*-trnfM*(*CAU*), and *psbE-petL*). Maximum Likelihood (ML) phylogenetic analyses based on the complete plastome and *ycf1* gene datasets consistently resolved the *Rosa* species into three major clades, with strong bootstrap support. In contrast, the ML tree based on *ITS* resolved species into four clades but showed lower bootstrap values, indicating reduced resolution compared to plastid datasets. Conclusions: Our findings underscore the value of plastome data in resolving phylogenetic relationships within the genus *Rosa*.

## 1. Introduction

The genus *Rosa* L., belonging to the Rosaceae Juss family, comprises approximately 200 species [1] primarily distributed across the Northern Hemisphere [2]. In Kazakhstan, the genus *Rosa* is represented by 25 species and is distributed throughout all regions of the country [3]. Among them, *Rosa acicularis* Lindl., *Rosa laxa* Retz., and *Rosa spinosissima* L. are widely distributed in the territory of the country, while *Rosa iliensis* Chrshan. is an endemic species [4]. The most recent classification of the genus *Rosa* was proposed by Wissemann (2017) [5], based on the earlier framework of Rehder (1940) [6]. This classification divides the genus into four subgenera: *Rosa*, *Hulthemia* (Dumort.) Focke, *Platyrhodon* (Hurst) Rehder, and *Hesperhodos* Cockerell. The largest subgenus, *Rosa*, is further divided into ten sections: *Banksianae* Lindl., *Bracteatae* Thory, *Caninae* (DC.) Ser., *Carolinae* Crép., *Gallicanae* (DC.) Ser., *Indicae* Thory, *Laevigatae* Thory, *Pimpinellifoliae* (DC.) Ser., *Rosa* (=Cinnamomeae (DC.) Ser.), and *Synstylae* DC [5,6].

Numerous phylogenetic studies have been conducted to clarify the taxonomy of the genus *Rosa* using molecular genetic approaches. For example, *Rosa* phylogenetic relationships have been investigated based on various genetic markers, including *ITS*-1 and the *atpB-rbcL* intergenic spacer [1], *trnL*, *trnG*, and the *psbA-trnH* intergenic spacer [7], as well as *rpl16*, *trnL-F*, and *atpB-rbcL* [8]. More recently, Debray et al. (2022) [9] employed phylogenomic analyses using 96 informative single-copy orthologous tags to resolve relationships within the genus. Furthermore, the genetic diversity and population structure of different *Rosa* species have been extensively investigated using a variety of molecular markers. Among these, randomly amplified polymorphic DNA (RAPD) [10,11], amplified fragment length polymorphism (AFLP) [12,13], simple sequence repeats (SSRs) [14,15,16], and single nucleotide polymorphisms (SNPs) [17,18] have been widely applied, offering valuable insights into intra- and interspecific genetic variation. Despite the studies that have been conducted, the assessment of phylogenetic relationships is often complicated by spontaneous hybridization events, which contribute to the formation of new species [19]. For instance, *R. spinosissima* has been proposed to have a hybrid origin [20]. In various phylogenetic studies, this species is positioned between *Rosa* and *Pimpinellifoliae* sections [7,9], suggesting its intermediate genetic background. The taxonomy of the genus remains challenging and continues to be a subject of debate and uncertainty.

Complete plastome sequences have also been employed to assess the phylogenetic relationships among *Rosa* species [21,22,23,24]. The highly informative coding regions *ndhF*, *ycf1 ycf3-trnS*, *trnT-trnL*, and *psbE-petL* have been identified within the plastomes of *Rosa* species and proposed as potential DNA barcoding markers for future phylogenetic studies [21,24]. It is well established that plastomes are a primary source of informative DNA barcoding markers, which are widely employed in phylogenetic analyses across a broad range of plant taxa [25,26]. In addition to their phylogenetic applications, plastomes also facilitate the identification of simple sequence repeat (SSR) markers, which are valuable tools in population genetic studies [27,28].

Plastomes of most angiosperms exhibit a conserved quadripartite structure consisting of a large single-copy (LSC) region, a small single-copy (SSC) region, and a pair of inverted repeats (IRs) [29]. The gene content and structural organization of plastomes are highly conserved, with genome sizes typically ranging from 120 to 160 kilobases [30,31]. With the advancement of sequencing technologies, plastome sequencing has become faster and more accessible, leading to its widespread application in phylogenetic studies [32,33].

Species of the genus *Rosa* are predominantly shrubs valued for their nutritional [34], ornamental [35], and medicinal [36] properties. Moreover, the fruits of *Rosa* species are widely used in food products such as jams, jellies, and herbal teas [37] and are well known for their high vitamin C content [38]. Phenolic compounds play a significant role in the activities of antidiabetic, anti-hypertensive, anti-tumor, anti-atherosclerotic, anti-inflammatory, and anti-aging properties [39,40,41,42]. Several studies [43,44,45] have investigated phenolic compounds from various parts of the Rosa species, with a particular focus on their chemical composition and antioxidant properties. Among these species, *R. acicularis* is a notable plant species for its rich diversity of phenolic compounds, which are distributed throughout its leaves, flowers, roots, and fruits. Ellagitannins and flavonoid glycosides are particularly abundant in this species [46]. Notably, leaf extracts of *R. acicularis* have demonstrated promising antidiabetic potential [46]. The chemical composition of four different extracts from *R. laxa* was quantitatively analyzed [47]. The results revealed that the aqueous extract exhibited the strongest antioxidant capacity and the highest contents of total triterpene acids, flavonoids, and polyphenols. These findings support the potential application of *R. laxa* in disease prevention and therapeutic strategies [47]. The qualitative and quantitative composition of anthocyanins in *R. spinosissima* fruit has been identified, highlighting its potential as a valuable natural source of anthocyanins for medicinal applications [48]. The composition of flower volatiles and seed fatty acids in *R. iliensis* populations from the Almaty Region in Kazakhstan was investigated [49]. A high abundance of oxygenated monoterpenes characterized the flower volatiles. In the seeds, linoleic, α-linolenic, and oleic acids were identified as the major fatty acid constituents [49]. The findings highlight that the *Rosa* species in this study (*R. acicularis*, *R. iliensis*, *R. laxa*, and *R. spinosissima*) are promising sources of bioactive compounds, underscoring their potential as valuable resources for medicinal applications.

In this study, we sequenced and assembled the complete plastomes of four *Rosa* species—*R. acicularis*, *R. iliensis*, *R. laxa*, and *R. spinosissima*—using next-generation sequencing technologies. The four species studied are dominant components of plant communities [3] in various regions of the country, possess high medicinal value [46,47,48,49], and have not been previously studied in Kazakhstan using plastome data. The complete plastomes of *R. iliensis* and *R. laxa* were sequenced for the first time in this study. The main objectives of this study were to (i) characterize the complete plastomes of the four *Rosa* species, (ii) perform comparative and phylogenetic analyses of these plastomes in conjunction with publicly available *Rosa* plastome sequences from the National Center for Biotechnology Information (NCBI) GenBank database, and (iii) identify additional molecular markers that may be informative for future phylogenetic and population genetic studies.

## 2. Materials and Methods

### 2.1. Plant Material Collection and DNA Extraction

**Figure 1:**
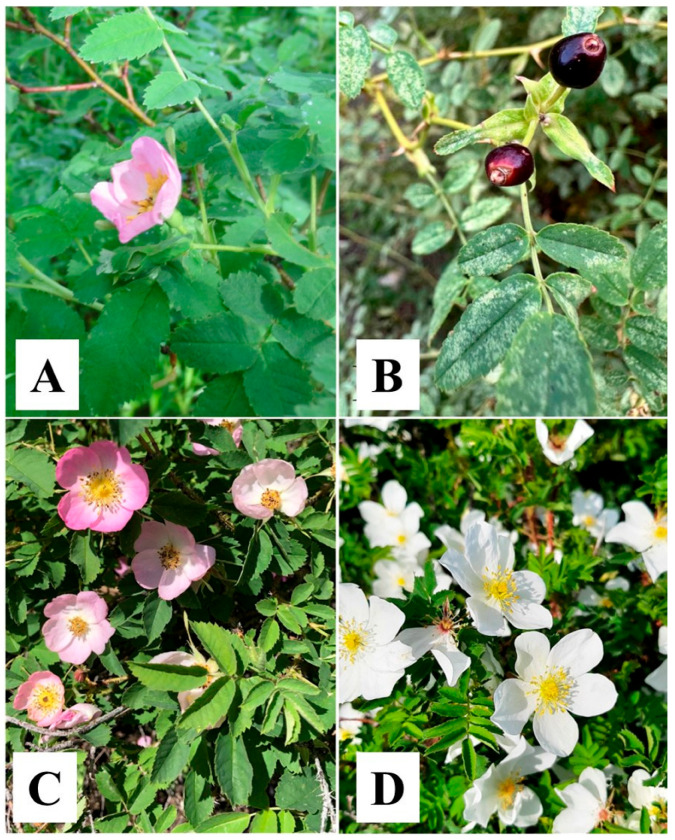
Pictures of the *Rosa acicularis* (**A**), *Rosa iliensis* (**B**), *Rosa laxa* (**C**), and *Rosa spinosissima* (**D**) species in nature.

Fresh leaf samples of *R. acicularis* were collected from the Karaganda Region, *R. iliensis* and *R. laxa* from the Mangystau Region, and *R. spinosissima* from the Western Altai Region (Figure 1). The collected fresh leaves were immediately dried in silica gel. Dried leaf material was subsequently used for total genomic DNA extraction using the DNeasy Plant Mini Kit (Qiagen, Valencia, CA, USA), following the manufacturer’s protocol. The concentration and quality of the extracted DNA were assessed using a NanoDrop 2000 spectrophotometer (Thermo Fisher Scientific, Wilmington, DE, USA) and by agarose gel electrophoresis.

### 2.2. PCR Amplification and Sequencing of the Internal Transcribed Spacer

The internal transcribed spacer (*ITS*) region of nuclear ribosomal DNA was utilized for phylogenetic analysis. Amplification of the *ITS* region was performed using the primers (Table 1) as described by White et al. (1990) [50].

The polymerase chain reaction (PCR) was conducted in a total reaction volume of 25 µL, containing 10× reaction buffer, 25 mM MgCl_2_, 2 µM of each primer, 4 mM of each dNTP, 1.6 units of Taq DNA polymerase, and 100 ng of genomic DNA. The volume was adjusted to 25 µL with nuclease-free water. The PCR was carried out using a SimpliAmp™ thermal cycler (Thermo Fisher Scientific, Carlsbad, CA, USA) under the following thermocycling conditions: initial denaturation at 94 °C for 3 min, followed by 40 cycles of denaturation at 94 °C for 30 s, annealing at 58 °C for 45 s, and extension at 72 °C for 1 min and 30 s, with a final extension at 72 °C for 10 min. PCR products were separated on a 1.5% agarose gel and subsequently purified using the ULTRAPrep^®^ Agarose Gel Extraction Mini Prep Kit (AHN Biotechnologie GmbH, Nordhausen, Germany), following the manufacturer’s protocol. Purified amplicons were used for sequencing with both forward and reverse primers using the BigDye^®^ Terminator Cycle Sequencing Kit (Applied Biosystems, Foster City, CA, USA). Sequencing was performed on an ABI 3130 Genetic Analyzer (Applied Biosystems, USA). Nucleotide sequences of the *ITS* of four *Rosa* species were deposited to the NCBI GenBank (https://www.ncbi.nlm.nih.gov/, accessed on 14 June 2025) under accession numbers PV789381, PV789382, PV789383, and PV789384.

### 2.3. Plastome Sequencing, Assembly, and Annotation

High-quality DNA samples met quality control standards for paired-end library preparation with the TruSeq Nano DNA Library Prep Kit (Illumina Inc., San Diego, CA, USA). Paired-end sequencing of the four *Rosa* species was performed on the Illumina NovaSeq 6000 platform (Illumina Inc., San Diego, CA, USA) at Macrogen Inc. (Seoul, Republic of Korea). The quality of the raw sequencing reads was evaluated using FastQC (https://www.bioinformatics.babraham.ac.uk/projects/fastqc/, accessed on 10 February 2025), and adapter sequences were removed using Trimmomatic v0.36 [51]. Cleaned reads were assembled de novo using NOVOPlasty v4.3.3 [52]. Plastome annotations were conducted using GeSeq [53], and the results were manually curated by comparison with reference sequences available in the NCBI GenBank. The annotated plastome sequences of *R. acicularis*, *R. iliensis*, *R. laxa*, and *R. spinosissima* have been deposited in GenBank (https://www.ncbi.nlm.nih.gov/, accessed on 10 March 2025) under accession numbers PV330080, PV330081, PV330082, and PV330083, respectively. Circular plastome maps were generated using Organellar Genome DRAW (OGDRAW) v1.3.1 [54].

### 2.4. Identification of the Simple Sequence Repeats 

To identify simple sequence repeat (SSR or microsatellite) elements within the plastomes of four *Rosa* species (*R. acicularis*, *R. iliensis*, *R. laxa*, and *R. spinosissima*), the MISA (Microsatellite Identification Tool) online tool was utilized [55]. The minimum repeat thresholds were set to 8 for mononucleotide repeats, 4 for di- and trinucleotide repeats, and 3 for tetra-, penta-, and hexanucleotide repeats. All identified SSR loci were manually inspected to ensure accuracy; redundant or overlapping entries were removed from the final dataset.

### 2.5. Plastome Comparison

A comparative analysis of the plastomes from the four Rosa species was performed using the mVISTA tool [56] with the Shuffle-LAGAN mode. The plastomes of *R. graciliflora* (OQ992658) and *R. acicularis* (MK714016) were used as reference sequences. To further investigate the boundaries of the inverted repeat (IR) regions, the junction sites of the four plastomes were analyzed using the IRscope tool [57], employing the same reference genomes, *R. graciliflora* (OQ992658) and *R. acicularis* (MK714016).

### 2.6. Sliding Window Analysis and Phylogenetic Analysis

For sliding window analysis and phylogenetic analysis, plastome sequences of the *Rosa* samples were aligned using Geneious Prime^®^ 2025.0.3. To evaluate nucleotide diversity, a sliding window analysis was conducted in DnaSP v6 [58], using a window size of 600 bp and a step size of 200 bp. This approach enabled the identification of highly variable regions across the aligned plastomes. For the phylogenetic analysis, three datasets were utilized: complete plastome nucleotide sequences, *ycf1* gene sequences, and nuclear ribosomal internal transcribed spacer (*ITS*) sequences. Phylogenetic trees were reconstructed using the Maximum Likelihood (ML) method, implemented in IQ-TREE v2.2.2.6 [59]. The best-fit nucleotide substitution models, selected based on the Bayesian Information Criterion (BIC), were TVM+F+I+R4 for the complete plastomes, TVM+F+G4 for the *ycf1* gene, and TIM+F+G4 for the *ITS* dataset. The resulting ML trees were visualized using FigTree v1.4.4 (http://tree.bio.ed.ac.uk/software/figtree/, accessed on 27 February 2025). The number of samples used in the phylogenetic analyses, based on different data sets, is provided in Table S1. The section names were assigned following Wisseman (2017) [5] and Rehder (1940) [6], while subclade designations were based on the work proposed by Debray et al. (2022) [9].

## 3. Results

### 3.1. General Characteristics of the Four Rosa Plastomes

**Figure 2:**
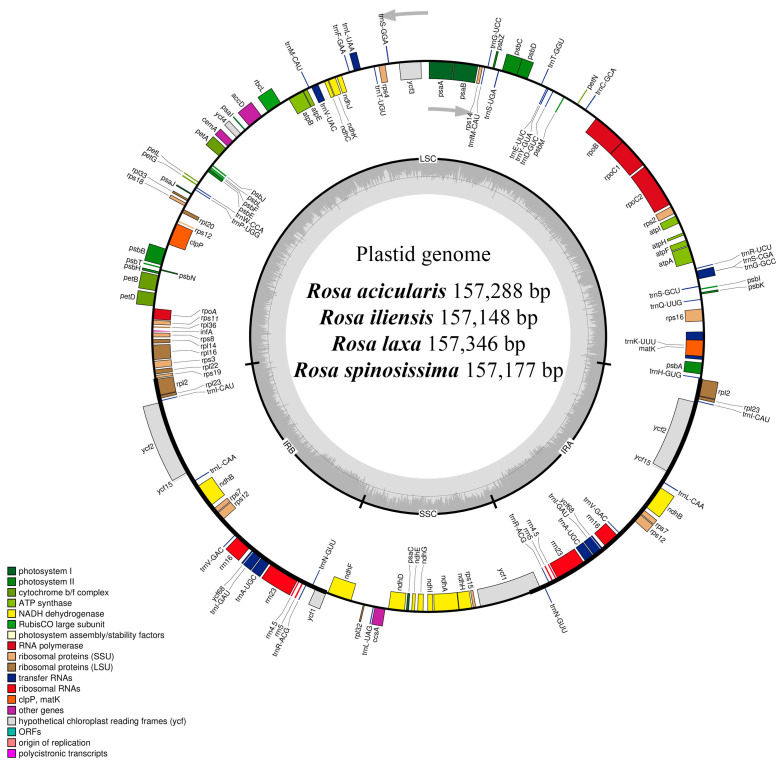
The plastome map of the *Rosa acicularis*, *Rosa iliensis*, *Rosa laxa*, and *Rosa spinosissima* species. The plastome map highlights the large single-copy (LSC) region, the small single-copy (SSC) region, and the inverted repeat regions (IRA and IRB). Genes located outside the outer circle are transcribed in a counterclockwise direction, whereas those within the circle are transcribed in a clockwise direction. The inner circle illustrates the content of GC (darker gray) and AT (lighter gray). Genes are color-coded based on their functional categories.

The complete plastome sizes of *R. acicularis*, *R. iliensis*, *R. laxa*, and *R. spinosissima* were determined to be 157,288 bp, 157,148 bp, 157,346 bp, and 157,177 bp, respectively (Table 2). All sequenced plastomes exhibited the typical quadripartite structure (Figure 2), comprising a large single-copy (LSC) region ranging from 86,269 (*R. spinosissima*) to 86,461 bp (*R. laxa*), a small single-copy (SSC) region varying between 18,778 (*R. iliensis*) and 18,791 bp (*R. acicularis* and *R. laxa*), and two inverted repeat (IR) regions ranging from 26,047 (*R. laxa*) to 26,062 bp (*R. spinosissima*). The overall GC content was consistent across all species, measured at 37.20% (Table 2).

The number of annotated genes was identical across all four plastomes, with a total of 136 genes identified. These included 90 protein-coding genes, 38 tRNA genes, and eight rRNA genes, including duplicated copies (Table 2). Twenty genes were duplicated within the IR regions, comprising nine protein-coding genes (*ndhB*, *rpl2*, *rpl23*, *rps7*, *rps12*, *ycf1*, *ycf2*, *ycf15*, and *ycf68*), seven tRNA genes (*trnA-UGC*, *trnI-CAU*, *trnI-GAU*, *trnL-CAA*, *trnN-GUU*, *trnR-ACG*, and *trnV-GAC*), and all four rRNA genes (*rrn4.5*, *rrn5*, *rrn16*, and *rrn23*). Among the annotated genes, seven tRNA genes (*trnA-UGC*, *trnG-GCC*, *trnI-GAU*, *trnK-UUU*, *trnL-UAA*, *trnS-CGA*, and *trnV-UAC*) and 11 protein-coding genes (*rps12*, *rps16*, *rpl2*, *rpl16*, *rpoC1*, *ndhA*, *ndhB*, *petD*, *clpP*, and *ycf3*) contained introns. The protein-coding genes *rps12*, *clpP*, and *ycf3* each harbored two introns, while the others contained a single intron (Table 3).

### 3.2. Simple Sequence Repeat Analysis

Simple sequence repeats (SSRs), also known as microsatellites, were identified within the plastome sequences of the four *Rosa* species. A total of 905 SSRs were detected, with individual counts of 224, 227, 225, and 229 in *R. acicularis*, *R. iliensis*, *R. laxa*, and *R. spinosissima*, respectively. The identified SSRs ranged from mono- to hexanucleotide repeats, although not all six types were present in each species. Mononucleotide repeats were the most abundant, accounting for 601 SSRs (66.41%), and ranged from 147 in *R. acicularis* to 152 in *R. spinosissima*. These were predominantly A/T repeats across all plastomes. Dinucleotide repeats (AT/AT, AG/CT, and AC/GT) were the second most common, comprising 223 SSRs (24.64%), and were mainly represented by AT/AT motifs. Their abundance ranged from 55 in *R. spinosissima* to 56 in *R. acicularis*, *R. iliensis*, and *R. laxa*. Trinucleotide SSRs (AAT/ATT and AGC/CTG) and tetranucleotide SSRs (AAAT/ATTT, AATT/AATT, ACAT/ATGT, and ACCT/AGGT) were less frequent, with 27 (2.98%) and 43 (4.75%), respectively. Pentanucleotide repeats (AATAT/ATATT and AAAAG/CTTTT) were rare, with only three (0.33%) identified across three of the species, absent in *R. iliensis*. Hexanucleotide repeats (AAGTAG/ACTTCT) were found in all four plastomes, totaling eight repeats (0.88%) (Table 4). Detailed information on the identified SSRs is provided in Supplementary File S1.

### 3.3. Comparative Analysis of the Plastomes of Four Rosa Species

**Figure 3:**
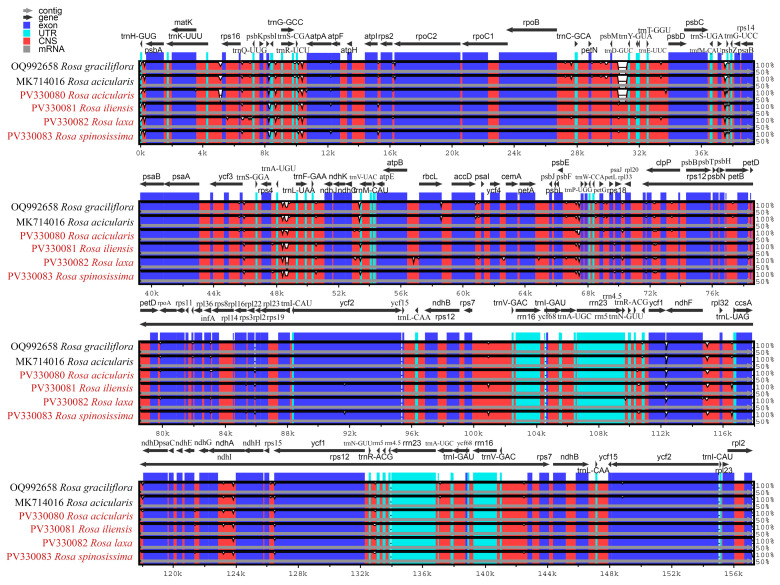
Comparison of the plastomes of *Rosa* species using mVISTA. The plastomes of *Rosa graciliflora* (OQ992658) and *Rosa acicularis* (MK714016) were used as the references. Species names highlighted in red represent those sequenced in this study. Coding regions (exons), untranslated regions (UTRs), and conserved non-coding sequences (CNSs) are represented in purple, blue, and red, respectively. The lower and upper horizontal axis indicates genome coordinates in kilobases (kb) and gene annotations, respectively. The vertical scale represents percent identity, ranging from 50% to 100%.

To assess sequence conservation and divergence across plastomes of four *Rosa* species, a mVISTA-based alignment was performed using the complete plastomes of *R. graciliflora* (OQ992658) and *R. acicularis* (MK714016) from NCBI GenBank as the references. The results revealed that the LSC and SSC regions are more variable than the IR regions, which showed higher sequence conservation across the examined *Rosa* samples. Overall, the alignment demonstrates a high degree of sequence conservation across the plastomes of *Rosa* samples, with notable divergence localized to non-coding regions (Figure 3).

**Figure 4:**
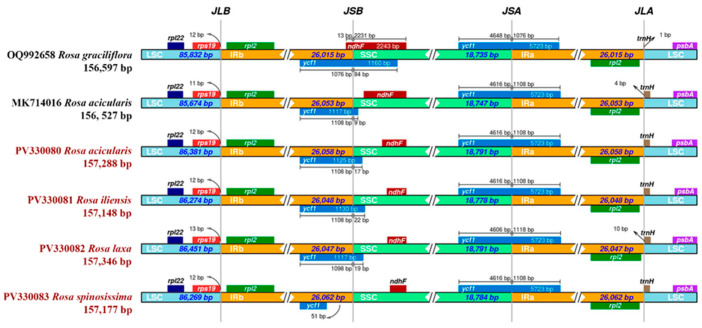
Comparison of junctions between large single-copy (LSC), small single-copy (SSC), and inverted repeat regions (IRa and IRb). Junctions are labeled as follows: JLB (LSC/IRb), JSB (IRb/SSC), JSA (SSC/IRa), and JLA (IRa/LSC). Samples highlighted in red represent the species sequenced in this study.

Next, the expansion and contraction of the IR regions at the LSC/IR/SSC junctions were analyzed using the reference plastomes of *R. graciliflora* (OQ992658) and *R. acicularis* (MK714016). The results revealed that the overall structure of the plastomes is conserved among the analyzed *Rosa* samples. However, minor differences in the positioning of boundary genes, such as *rps19*, *ycf1*, *ndhF*, and *trnH*, reflect subtle expansions or contractions of the IR regions. At the LSC/IRb junction (JLB), the rps19 gene consistently overlaps with the IRb region in all six samples, with an overlap ranging from 11 to 13 bp. The *ndhF* gene is located at the IRb/SSC junction (JSB) only in *R. graciliflora* (OQ992658) and is absent at this junction in the remaining five plastomes. The *ycf1* gene spans the SSC/IRb junction (JSB) in five genomes but is absent at this boundary in *R. spinosissima* (PV330083). Additionally, a duplicated copy of the *ycf1* gene is present at the IRa/SSC (JSA) junction in all six plastomes. The IRa/LSC junction (JLA) is relatively conserved, with the *rpl2* gene located entirely within the IRa region, while the *trnH* gene is positioned downstream in the LSC, showing slight positional variation ranging from 1 to 10 bp. In terms of regional lengths, the LSC region ranges from 85,674 bp in *R. acicularis* (MK714016) to 86,451 bp in *R. laxa* (PV330082). The SSC region varies from 18,735 bp in *R. graciliflora* (OQ992658) to 18,791 bp in *R. acicularis* (PV330080) and *R. laxa* (PV330082). The length of each IR region (IRa and IRb) ranges from 26,015 bp in *R. graciliflora* to 26,062 bp in the *R. spinosissima* (PV330083) plastome (Figure 4).

### 3.4. Nucleotide Diversity Analysis by Sliding Window

**Figure 5:**
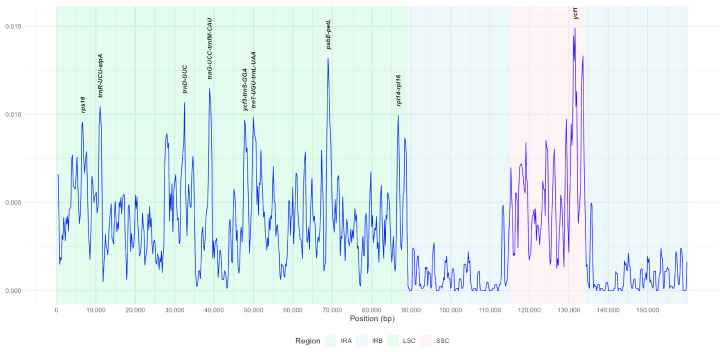
Nucleotide diversity (Pi) across the plastomes of 26 *Rosa* samples, estimated using sliding window analysis. The Pi values for each window are plotted on the *Y*-axis, while the corresponding positions are represented on the *X*-axis.

A nucleotide diversity analysis was conducted on the complete plastome sequences of 26 *Rosa* samples, including four studied species. The nucleotide diversity (Pi) values ranged from 0 to 0.01488. Nine highly variable regions were identified, exhibiting relatively elevated Pi values. These included two coding regions (*rps16* and *ycf1*) and seven intergenic regions (*ycf3-trnS*(*GGA*), *trnT*(*UGU*)*-trnL*(*UAA*), *rpl14-rpl16*, *trnR*(*UCU*)*-atpA*, *trnD*(*GUC*), *trnG*(*UCC*)*-trnfM*(*CAU*), and *psbE-petL*). The highest nucleotide diversity (Pi = 0.01488) was observed in the *ycf1* gene region of the SSC region. Overall, the LSC and SSC regions exhibited the greatest nucleotide variability, with the LSC region showing higher nucleotide diversity than the SSC region (Figure 5).

### 3.5. Phylogenetic Analysis

**Figure 6:**
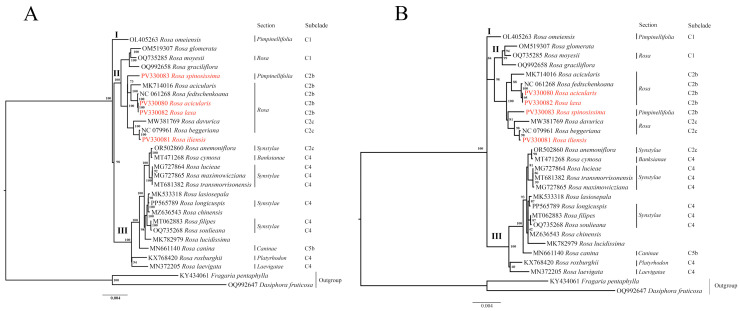
Maximum Likelihood phylogenetic trees based on (**A**) nucleotide sequences of the complete plastome and (**B**) the *ycf1* gene. Species names highlighted in red represent sequenced samples from this study. Section names are provided according to the classification of Wissemann (2017) [5] and Rehder (1940) [6], and subclade designations (C1, C2b, C4, and C5b) follow the subclade structure inferred from Debray et al. (2022) [9]. Roman numerals (I–III) indicate major clades. Numbers at the nodes represent bootstrap support values.

**Figure 7:**
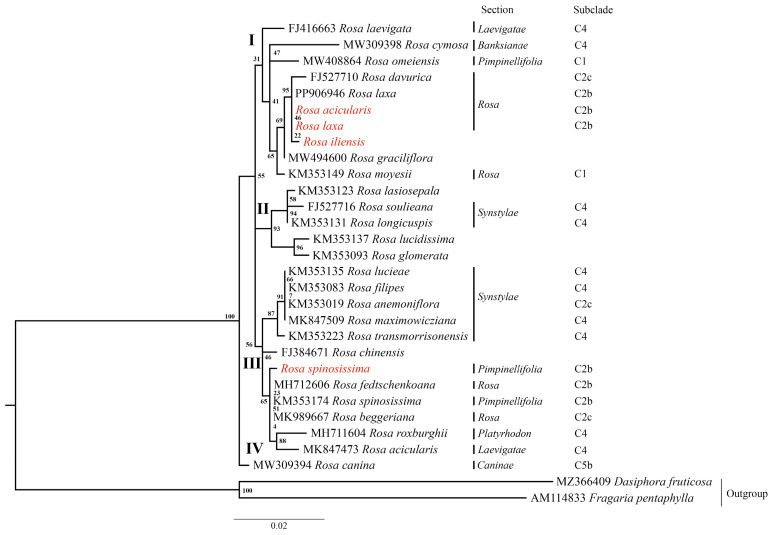
Maximum Likelihood phylogenetic tree based on nucleotide sequences of the nuclear ribosomal internal transcribed spacer. Species names highlighted in red represent sequenced samples from this study. Section names are provided according to the classification of Wissemann (2017) [5] and Rehder (1940) [6], and subclade designations (C1, C2b, C4, and C5b) follow the subclade structure inferred from Debray et al. (2022) [9]. Roman numerals (I–IV) indicate major clades. Numbers at the nodes represent bootstrap support values.

Phylogenetic trees were reconstructed using the Maximum Likelihood (ML) method based on three datasets: (1) nucleotide sequences of the complete plastome (Figure 6A), (2) nucleotide sequences of the *ycf1* gene (Figure 6B), and (3) nucleotide sequences of the nuclear ribosomal internal transcribed spacer (ITS) (Figure 7). *Fragaria pentaphylla* and *Dasiphora fruticosa* were used as outgroups in all analyses. Based on the complete plastome and *ycf1* gene datasets, the ML trees consistently resolved the *Rosa* species into three major clades (I, II, and III), with strong bootstrap support at most nodes. Clade I included *R. omeiensis* from the *Pimpinellifoliae* section. Clade II comprised species from sections *Pimpinellifoliae* and *Rosa*, forming subclades C1, C2b, and C2c. Notably, the species analyzed in this study, *R. spinosissima*, *R. acicularis*, *R. laxa*, and *R. iliensis*, are grouped within subclade C2b. Clade III was the most taxonomically diverse, including species from sections *Synstylae*, *Banksianae*, *Caninae*, *Laevigatae*, and *Platyrhodon*, primarily corresponding to clades C4 and C5b. An exception was *R. anemoniiflora*, which grouped within the subclade C2c (Figure 6). In contrast, the ML phylogenetic tree based on the ITS nucleotide sequences (Figure 7) resolved the species into four clades (I, II, III, and IV). The studied species *R. acicularis*, *R. iliensis*, and *R. laxa* were grouped in Clade I, along with *R. davurica* and *R. laxa* from GenBank, corresponding to subclade C2b. Meanwhile, *R. spinosissima* (C2b, this study) grouped in Clade III with GenBank samples of *R. fedschenkoana*, *R. spinosissima*, *R. beggeriana* (all C2b), *R. roxburghii* (C4), and *R. acicularis* (C4). The ITS tree showed lower bootstrap support than the plastome and *ycf1* gene trees, indicating reduced resolution in the nuclear dataset (Figure 7).

Overall, the phylogenetic analysis based on the complete plastome and *ycf1* gene nucleotide sequences revealed clear sectional affiliations and evolutionary relationships among the *Rosa* species, with strong bootstrap support indicating the robustness of the inferred topology.

## 4. Discussion

The development of low-cost, high-throughput sequencing technologies has greatly accelerated the sequencing of plant plastomes. In this study, we sequenced, assembled, and conducted a comparative analysis of the complete plastomes of four *Rosa* species from Kazakhstan: *R. acicularis*, *R. iliensis*, *R. laxa*, and *R. spinosissima* (Figure 1), using next-generation sequencing technologies. Consistent with the typical structure observed in most angiosperms [60,61], the plastomes of the studied *Rosa* species exhibited a quadripartite organization, comprising LSC and SSC regions, and two IR regions (Figure 2). The genome sizes ranged from 157,148 bp in *R. iliensis* to 157,346 bp in *R. laxa* (Table 2), closely aligning with the plastome lengths reported for *R. glomerata* Rehder & E.H. Wilson (157,064 bp) [62], *R. praelucens* Bijh. (157,186 bp) [63], and *R. minutifolia* Engelm. (157,396 bp) [64]. The GC content across all four species was 37.20%, which is consistent with the GC content of the *R. xanthina* Lindl. plastome (37.20%) [65].

Each genome contained 136 annotated genes, including 90 protein-coding genes, 38 tRNA genes, and eight rRNA genes. Twenty of these genes were duplicated within the IR regions: nine protein-coding genes (*ndhB*, *rpl2*, *rpl23*, *rps7*, *rps12*, *ycf1*, *ycf2*, *ycf15*, and *ycf68*), seven tRNA genes (*trnA-UGC*, *trnI-CAU*, *trnI-GAU*, *trnL-CAA*, *trnN-GUU*, *trnR-ACG*, and *trnV-GAC*), and all four rRNA genes (*rrn4.5*, *rrn5*, *rrn16*, and *rrn23*) (Table 3). The number of duplicated tRNA genes was consistent with previous reports from other *Rosa* species [63,66]. The plastomes of most land plants are highly conserved in gene content and structure [67]. However, certain genes exhibit high variability and have been entirely lost or pseudogenized during the evolution of angiosperms [31]. Among these, the *infA* gene stands out as one of the most dynamic, and it is frequently lost from the plastome and functionally transferred to the nuclear genome [68]. The loss of the *infA* gene through pseudogenization has been detected in some Rosa plastomes [21,69] and is reported as a common occurrence among *Rosa* species belonging to the section *Synstylae*. Additionally, the *rps19* gene was also observed to be missing in some *Rosa* plastomes [69], further highlighting the variability of plastomes in this genus. The loss of *infA*, as well as other genes such as *accD*, *rpl22*, *rps19*, and several hypothetical open reading frame genes (*ycf*), has been previously reported across various angiosperm lineages [70,71,72,73]. These gene losses are often lineage-specific and may reflect functional gene transfer events or adaptive evolutionary pressures related to specific ecological conditions [74].

The mVISTA-based alignment of the plastomes revealed that the LSC and SSC regions exhibit greater sequence variability compared to the IR regions (Figure 3). This pattern is commonly observed in the plastomes of most angiosperms [75,76]. Overall, the alignment confirms a high level of sequence conservation among the studied *Rosa* plastomes, with the majority of sequence divergence occurring in non-coding regions. These findings are consistent with previous studies that reported similar patterns of variation and conservation in the plastomes of related taxa [77,78].

The expansion and contraction of the IR regions at the junctions with the LSC and SSC regions were analyzed using reference plastomes of *R. graciliflora* (OQ992658) and *R. acicularis* (MK714016) obtained from GenBank (Figure 4). The results demonstrate that the overall structure of the plastomes is conserved among the *Rosa* species examined, which aligns with previous findings on *Rosa* plastomes [21,24]. Notably, the *ycf1* gene spans the SSC/IRb junction (JSB) in five of the analyzed genomes; however, this gene is absent at the corresponding boundary in the plastome of *R. spinosissima* (PV330083), as reported in the present study. This observation is consistent with earlier reports on the plastomes of *R. roxburghii* and *R. odorata* var. *gigantea*, where similar variations at the SSC/IRb junction have been identified [79].

SSRs, also known as microsatellites, are well recognized for their high polymorphism [80] and are widely utilized in studies of plant population genetic diversity [81,82]. Previous research has demonstrated the potential and significance of plastid-derived SSRs for assessing genetic variation in species from the genera *Carya* [83], *Juglans* [84], and *Abies* [85]. In this study, we identified a total of 905 SSRs across the plastomes of four *Rosa* species: *R. acicularis*—224 SSRs, *R. iliensis*—227 SSRs, *R. laxa*—225 SSRs, and *R. spinosissima*—229 SSRs (Table 3). Most of these SSRs were mononucleotide repeats (66.41%) and were predominantly located in non-coding LSC and SSC regions. This pattern is consistent with previous findings that show SSRs are more commonly found in non-coding rather than coding regions of plastomes [76,86]. Most of the SSRs identified in this study were composed of A/T or AT/AT motifs, which align with patterns observed in the plastomes of other plant species [87,88]. These SSRs may serve as valuable molecular markers for future population genetic studies within *Rosa* species and could also be potentially transferable across related taxa.

DNA barcoding markers, in addition to SSRs, serve as essential tools for evaluating plant phylogenetic relationships [89] and facilitating species identification [90,91]. Barcodes derived from the plastome have proven particularly valuable in studies of plant molecular evolution across a wide range of plant taxa [92,93]. Despite their utility, the informativeness of commonly used barcoding regions varies significantly among different plant groups, and not all markers provide sufficient resolution for phylogenetic inference [94]. Therefore, identifying highly informative, taxon-specific DNA barcoding markers is critical for accurately resolving phylogenetic relationships and improving species delimitation within complex plant lineages. Consequently, we performed nucleotide diversity analysis to identify the polymorphic regions of the plastomes of *Rosa* species. Nine highly variable regions were identified, including two coding regions (*rps16* and *ycf1*) and seven intergenic regions (*ycf3-trnS*(*GGA*), *trnT*(*UGU*)*-trnL*(*UAA*), *rpl14-rpl16*, *trnR*(*UCU*)*-atpA*, *trnD*(*GUC*), *trnG*(*UCC*)*-trnfM*(*CAU*), and *psbE-petL*) (Figure 5). Consistent with previous research, the intergenic regions *trnT*(*UGU*)*-trnL*(*UAA*) [95], *psbE-petL* [74], *trnT-trnL* [96], and the coding region *ycf1* [97] have been identified in various Rosaceae species as hotspots of nucleotide diversity. In addition, the coding regions *rps16* (0,00957) and *ycf1* (0,01488) were also identified across the plastomes of various *Rosa* species [21,24]. Due to their high variability and coding, intergenic regions have been proposed as strong candidates for DNA barcoding, potentially improving species discrimination and resolving phylogenetic relationships within families and genera.

In this study, in addition to the complete plastome nucleotide sequences (Figure 6A) and nuclear ribosomal *ITS* region nucleotide sequences (Figure 7), we also incorporated the *ycf1* gene nucleotide sequences into our phylogenetic analysis (Figure 6B). The ML phylogenetic trees generated from the complete plastome and *ycf1* datasets revealed congruent topologies (Figure 6), each resolving *Rosa* species into three major clades (Clades I, II, and III) with robust bootstrap support across most nodes. These topologies align closely with the ML tree constructed using single-copy orthologous tags, as reported by Debray et al. (2022) [9], underscoring the reliability of both the complete plastome and the *ycf1* gene as effective molecular markers for resolving evolutionary relationships within the genus *Rosa*. In contrast, the ML phylogenetic tree constructed from *ITS* sequences (Figure 7) resolved the species into four clades (Clades I, II, III, and IV) but exhibited lower bootstrap support, indicating a reduced resolution power for the studied dataset. Although several reports on *Rosa* have employed *ITS* sequences [98,99], Wissemann and Ritz (2005) [1] have combined *ITS* with plastid markers, such as *atpB–rbcL*, to improve phylogenetic resolution. Additionally, several studies for phylogenetic resolution on different taxa have reported limitations of *ITS* sequences compared to complete plastomes [88,100]. Based on our findings and supporting evidence from the literature, we conclude that *ITS* sequences alone may not provide sufficient resolution to accurately delineate phylogenetic relationships among *Rosa* species. Instead, *ITS* should be used in combination with informative plastid markers, as recommended by earlier reports [21,24], to enhance the robustness of phylogenetic inference within this taxonomically complex genus *Rosa*.

Overall, our study demonstrates that plastome analyses offer valuable insights for future phylogenetic and taxonomic research within the genus *Rosa*. By comparing multiple datasets, including complete plastome sequences, the *ycf1* gene, and nuclear *ITS* sequences, we highlight the superior resolution provided by plastome data, particularly in delineating major clades and uncovering evolutionary relationships. Despite the study conducted, further research is needed, including a wider range of *Rosa* species and the integration of morphological data, to improve evolutionary inferences within the genus *Rosa*.

## 5. Conclusions

In this study, we sequenced, assembled, and conducted comparative analyses of the complete plastomes of four *Rosa* species from Kazakhstan—*R. acicularis*, *R. iliensis*, *R. laxa*, and *R. spinosissima*. Sliding window analysis identified the *ycf1* gene region as having a relatively high nucleotide diversity (Pi = 0.01488), highlighting its potential as a reliable DNA barcoding marker for the *Rosa* genus. Phylogenetic analyses based on the *ycf1* gene and complete plastome data provided superior resolution compared to nuclear *ITS* sequences, effectively delineating major clades. For *R. iliensis*, we propose the possible section name *Rosa*, based on the results of phylogenetic analyses. These findings underscore the value of plastome data, particularly the *ycf1* gene, for resolving taxonomic complexities within *Rosa*. The study’s results provide a valuable genomic resource for future phylogenetic and taxonomic investigations of the genus.

## Figures and Tables

**Table 1:** Information on primers used for amplification.

Primer	Sequence (5′-3′)	Direction	Temperature (°C)	Reference
ITS1nF	AGAAGTCGTAACAAGGTTTCCGTAGG	Forward	58	[50]
ITS4nR	TCCTCCGCTTATTGATATGC	Reverse	58	[50]

**Table 2:** Summary of plastome characteristics of four *Rosa* species.

Species	*Rosa acicularis*	*Rosa iliensis*	*Rosa laxa*	*Rosa spinosissima*
GenBank accession number	PV330080	PV330081	PV330082	PV330083
Genome size (bp)	157,288	157,148	157,346	157,177
LSC length (bp)	86,381	86,274	86,461	86,269
SSC length (bp)	18,791	18,778	18,791	18,784
IRA length (bp)	26,058	26,048	26,047	26,062
IRB length (bp)	26,058	26,048	26,047	26,062
Total GC content (%)	37.20	37.20	37.20	37.20
Number of total genes (unique)	136 (116)	136 (116)	136 (116)	136 (116)
Total protein-coding genes (unique)	90 (81)	90 (81)	90 (81)	90 (81)
Total tRNA genes (unique)	38 (31)	38 (31)	38 (31)	38 (31)
Total rRNA genes (unique)	8 (4)	8 (4)	8 (4)	8 (4)

**Table 3:** List of the annotated genes in the *Rosa acicularis*, *Rosa iliensis*, *Rosa laxa*, and *Rosa spinosissima* plastomes.

Group of Genes	Name of Genes
Self-replication	
Ribosomal RNA	*rrn4*.5 (×2), *rrn5* (×2), *rrn16* (×2), *rrn23* (×2)
Transfer RNA	*trnA-UGC* * (×2), *trnC-GCA*, *trnD-GUC*, *trnE-UUC*, *trnF-GAA*, *trnfM-CAU*, *trnG-GCC* *, *trnG-UCC*, *trnH-GUG*, *trnI-CAU* (×2), *trnI-GAU* * (×2), *trnK-UUU* *, *trnL-CAA* (×2), *trnL-UAA* *, *trnL-UAG*, *trnM-CAU*, *trnN-GUU* (×2), *trnP-UGG*, *trnQ-UUG*, *trnR-ACG* (×2), *trnR-UCU*, *trnS-CGA* *, *trnS-GCU*, *trnS-GGA*, *trnS-UGA*, *trnT-GGU*, *trnT-UGU*, *trnV-GAC* (×2), *trnV-UAC* *, *trnW-CCA*, *trnY-GUA*
Small subunit of ribosome	*rps2*, *rps3*, *rps4*, *rps7* (×2), *rps8*, *rps11*, *rps12* ** (×2), *rps14*, *rps15*, *rps16* *, *rps18*, *rps19*
Large subunit of ribosome	*rpl2* * (×2), *rpl14*, *rpl16* *, *rpl20*, *rpl22*, *rpl23* (×2), *rpl32*, *rpl33*, *rpl36*
RNA polymerase	*rpoA*, *rpoB*, *rpoC1* *, *rpoC2*
Translation initiation factor	*infA*
Photosynthesis	
ATP synthase	*atpA*, *atpB*, *atpE*, *atpF*, *atpH*, *atpI*
NADH dehydrogenase	*ndhA* *, *ndhB* * (×2), *ndhC*, *ndhD*, *ndhE*, *ndhF*, *ndhG*, *ndhH*, *ndhI*, *ndhJ*, *ndhK*
Subunits of cytochrome	*petA*, *petB* *, *petD* *, *petG*, *petL*, *petN*
Photosystem I	*psaA*, *psaB*, *psaC*, *psaI*, *psaJ*
Photosystem II	*psbA*, *psbB*, *psbC*, *psbD*, *psbE*, *psbF*, *psbH*, *psbI*, *psbJ*, *psbK*, *psbL*, *psbM*, *psbN*, *psbT*, *psbZ*
Rubisco	*rbcL*
Other genes	
Maturase	*matK*
Protease	*clpP* **
Envelope membrane protein	*cemA*
Subunit of acetyl-CoA-carboxylase	*accD*
C-type cytochrome synthesis gene	*ccsA*
Genes of unknown function	
Conserved hypothetical chloroplast ORF	*ycf1* (×2), *ycf2* (×2), *ycf3* **, *ycf4*, *ycf15* (×2), *ycf68* (×2)

A single asterisk (*) indicates genes containing one intron, while a double asterisk (**) indicates genes with two introns. Genes marked with (×2) are duplicated and located in the inverted repeat (IR) regions.

**Table 4:** The type and number of identified simple sequence repeats in four *Rosa* plastomes.

Type	Repeat Unit	*Rosa acicularis*	*Rosa iliensis*	*Rosa laxa*	*Rosa spinosissima*	Total	%
Mono-	A/T	138	144	138	143	563	66.41
	C/G	9	10	10	9	38	
Di-	AT/AT	39	39	39	38	155	24.64
	AG/CT	16	16	16	16	64	
	AC/GT	1	1	1	1	4	
Tri-	AAT/ATT	7	5	7	7	26	2.98
	AGC/CTG	0	0	0	1	1	
Tetra-	AAAT/ATTT	6	6	6	6	24	4.75
	AATT/AATT	2	1	2	2	7	
	ACAT/ATGT	1	1	1	1	4	
	ACCT/AGGT	2	2	2	2	8	
Penta-	AATAT/ATATT	1	0	1	0	2	0.33
	AAAAG/CTTTT	0	0	0	1	1	
Hexa-	AAGTAG/ACTTCT	2	2	2	2	8	0.88
Total		224	227	225	229	905	100.00

## Data Availability

Plastome data are available in the National Center for Biotechnology Information (NCBI) database under accession numbers PV330080, PV330081, PV330082, and PV330083. Nucleotide sequences of the *ITS* were also deposited in the NCBI under accession numbers PV789381, PV789382, PV789383, and PV789384.

## Supplementary Materials

The following supporting information can be downloaded at https://www.mdpi.com/article/10.3390/genes16080852/s1: Supplementary File S1: Information on the identified SSRs from the plastomes of four *Rosa* species; Table S1: The number of samples used in the phylogenetic analyses based on different data sets.

## References

[1] Wissemann V., Ritz C.M. (2005). The genus *Rosa* (Rosoideae, Rosaceae) revisited: Molecular analysis of nrITS-1 and *atpB–rbcL* intergenic spacer (IGS) versus conventional taxonomy. Bot. J. Linn. Soc..

[2] Rehder A. (1949). Bibliography of Cultivated Trees and Shrubs Hardy in the Cooler Temperate Regions of the Northern Hemisphere.

[3] Pavlov N.V. (1961). Flora of Kazakhstan.

[4] Childibayeva A., Ametov A., Kurbatova N.V., Akhmetova A., Tynybekov B.M., Mukanova G.A. (2022). Structural characteristics of *Rosa iliensis* Chrshan. under conditions of the floodplains of the Rivers Ili and Sharyn. J. Ecol. Eng..

[5] Wissemann V. (2017). Conventional Taxonomy (Wild Roses). https://www.researchgate.net/publication/312015123_Conventional_Taxonomy_Wild_Roses.

[6] Rehder A. (1940). Rosa L. In Manual of Cultivated Trees and Shrubs Hardy in North America.

[7] Fougère-Danezan M., Joly S., Bruneau A., Gao X.F., Zhang L.B. (2015). Phylogeny and biogeography of wild roses with specific attention to polyploids. Ann. Bot..

[8] Liu C., Wang G., Wang H., Xia T., Zhang S., Wang Q., Fang Y. (2015). Phylogenetic relationships in the genus *Rosa* revisited based on rpl16, trnL-F, and atpB–rbcL sequences. HortScience.

[9] Debray K., Le Paslier M.C., Bérard A., Thouroude T., Michel G., Marie-Magdelaine J., Bruneau A., Foucher F., Malécot V. (2022). Unveiling the patterns of reticulated evolutionary processes with phylogenomics: Hybridization and polyploidy in the genus. Rosa. Syst. Biol..

[10] Frederick C., Wagner A., Morvillo N. (2002). Randomly amplified polymorphic DNA (RAPD) analysis of the musk roses (*Rosa moschata*). Proc. Fla. State Hortic. Soc..

[11] Riaz A., Hameed M., Khan A.I., Younis A., Awan F.S. (2011). Assessment of biodiversity based on morphological characteristics and RAPD markers among genotypes of wild rose species. Afr. J. Biotechnol..

[12] De Riek J., De Cock K., Smulders M.J.M., Nybom H. (2013). AFLP-based population structure analysis as a means to validate the complex taxonomy of dogroses (*Rosa* section *Caninae*). Mol. Phylogenet. Evol..

[13] Koopman W.J.M., Wissemann V., De Cock K., Van Huylenbroeck J., De Riek J., Sabatino G.J., Visser D., Vosman B., Ritz C.M., Maes B. (2008). AFLP markers as a tool to reconstruct complex relationships: A case study in Rosa (Rosaceae). Am. J. Bot..

[14] Akond M., Jin S., Wang X. (2012). Molecular characterization of selected wild species and miniature roses based on SSR markers. Sci. Hortic..

[15] Gaurav A.K., Namita, Raju D.V.S., Ramkumar M.K., Singh M.K., Singh B., Krishnan S.G., Panwar S., Sevanthi A.M. (2021). Genetic diversity analysis of wild and cultivated *Rosa* species of India using microsatellite markers and their comparison with morphology-based diversity. J. Plant Biochem. Biotechnol..

[16] Yang C., Ma Y., Cheng B., Zhou L., Yu C., Luo L., Pan H., Zhang Q. (2020). Molecular evidence for hybrid origin and phenotypic variation of Rosa section Chinenses. Genes.

[17] Heo M.S., Han K., Kwon J.K., Kang B.C. (2017). Development of SNP markers using genotyping-by-sequencing for cultivar identification in rose (Rosa hybrida). Hortic. Environ. Biotechnol..

[18] Xia A.N., Yang A.A., Meng X.S., Dong G.Z., Tang X.J., Lei S.M., Liu Y.G. (2022). Development and application of rose (Rosa chinensis Jacq.) SNP markers based on SLAF-seq technology. Genet. Resour. Crop Evol..

[19] Zhu Z.M., Gao X.F., Fougère-Danezan M. (2015). Phylogeny of Rosa sections Chinenses and Synstylae (Rosaceae) based on chloroplast and nuclear markers. Mol. Phylogenet. Evol..

[20] Roberts A.V. (1977). Relationship between species in the genus Rosa, section Pimpinellifoliae. Bot. J. Linn. Soc..

[21] Jeon J.H., Kim S.C. (2019). Comparative analysis of the complete chloroplast genome sequences of three closely related East-Asian wild roses (Rosa sect. Synstylae; Rosaceae). Genes.

[22] Yan H., Liu Y., Wu Z., Yi Y., Huang X. (2021). Phylogenetic relationships and characterization of the complete chloroplast genome of Rosa sterilis. Mitochondrial DNA B.

[23] Yin X., Liao B., Guo S., Liang C., Pei J., Xu J., Chen S. (2020). The chloroplasts genomic analyses of *Rosa laevigata*, *R. rugosa* and *R. canina*. Chin. Med..

[24] Gao C., Li T., Zhao X., Wu C., Zhang Q., Zhao X., Wu M., Lian Y., Li Z. (2023). Comparative analysis of the chloroplast genomes of *Rosa* species and RNA editing analysis. BMC Plant Biol..

[25] Appelhans M.S., Bayly M.J., Heslewood M.M., Groppo M., Verboom G.A., Forster P.I., Kallunki J.A., Duretto M.F. (2021). A new subfamily classification of the Citrus family (Rutaceae) based on six nuclear and plastid markers. Taxon.

[26] Zhang G., Feng C., Kou J., Han Y., Zhang Y., Xiao H. (2023). Phylogeny and divergence time estimation of the genus Didymodon (Pottiaceae) based on nuclear and chloroplast markers. J. Syst. Evol..

[27] Ebert D., Peakall R. (2009). Chloroplast simple sequence repeats (cpSSRs): Technical resources and recommendations for expanding cpSSR discovery and applications to a wide array of plant species. Mol. Ecol. Resour..

[28] Song S.L., Lim P.E., Phang S.M., Lee W.W., Hong D.D., Prathep A. (2014). Development of chloroplast simple sequence repeats (cpSSRs) for the intraspecific study of Gracilaria tenuistipitata (Gracilariales, Rhodophyta) from different populations. BMC Res. Notes.

[29] Kolodner R., Tewari K.K. (1979). Inverted repeats in chloroplast DNA from higher plants. Proc. Natl. Acad. Sci. USA.

[30] Wambugu P.W., Brozynska M., Furtado A., Waters D.L., Henry R.J. (2015). Relationships of wild and domesticated rices (Oryza AA genome species) based upon whole chloroplast genome sequences. Sci. Rep..

[31] Wicke S., Schneeweiss G.M., Depamphilis C.W., Müller K.F., Quandt D. (2011). The evolution of the plastid chromosome in land plants: Gene content, gene order, gene function. Plant Mol. Biol..

[32] Almerekova S., Yermagambetova M., Ivaschenko A., Turuspekov Y., Abugalieva S. (2024). Comparative analysis of plastome sequences of seven *Tulipa* L. (Liliaceae Juss.) species from section Kolpakowskianae Raamsd. ex Zonn and Veldk. Int. J. Mol. Sci..

[33] Nyamgerel N., Baasanmunkh S., Munkhtulga D., Tugsbilguun T., Oyuntsetseg B., Xiang C.L., Choi H.J. (2025). Characterization of the complete chloroplast genome of Dracocephalum ruyschiana (Lamiaceae) and its phylogenetic analysis. Korean J. Plant Taxon..

[34] Singh K., Gairola S. (2023). Nutritional Potential of Wild Edible Rose Hips in India for Food Security. Wild Food Plants for Zero Hunger and Resilient Agriculture.

[35] Pekamwar S.S., Kalyankar T.M., Jadhav A.C. (2013). Hibiscus rosa-sinensis: A review on ornamental plant. World J. Pharm. Pharm. Sci. (WJPPS).

[36] Takahashi N. (2025). Rose (Rosa sp.) More Than Just Beautiful: Exploring the Therapeutic Properties of the Rose Species. Advances in Medicinal and Aromatic Plants.

[37] Butkevičiūtė A., Urbštaitė R., Liaudanskas M., Obelevičius K., Janulis V. (2022). Phenolic content and antioxidant activity in fruit of the genus *Rosa* L. *Antioxidants*
**2022**, *11*, 912. Antioxidants.

[38] Chrubasik C., Roufogalis B.D., Müller-Ladner U., Chrubasik S. (2008). A systematic review on the *Rosa canina* effect and efficacy profiles. Phytother. Res..

[39] Akhtar N., Mirza B. (2018). Phytochemical analysis and comprehensive evaluation of antimicrobial and antioxidant properties of 61 medicinal plant species. Arab. J. Chem..

[40] Cagle P., Idassi O., Carpenter J., Minor R., Goktepe I., Martin P. (2012). Effect of rosehip (Rosa canina) extracts on human brain tumor cell proliferation and apoptosis. J. Cancer Ther..

[41] Jian C., Lu W., Tang X., Huang X., Chen H. (2015). Study on the anti-atherosclerosis effect of *Rosa roxburghii* Tratt. Asia-Pac. Tradit. Med..

[42] Wu H., Li M., Yang X., Wei Q., Sun L., Zhao J., Shang H. (2020). Extraction optimization, physicochemical properties and antioxidant and hypoglycemic activities of polysaccharides from roxburgh rose (*Rosa roxburghii* Tratt.) leaves. Int. J. Biol. Macromol..

[43] Liaudanskas M., Noreikienė I., Zymonė K., Juodytė R., Žvikas V., Janulis V. (2021). Composition and antioxidant activity of phenolic compounds in fruit of the genus *Rosa* L. Antioxidants.

[44] Chroho M., Bouymajane A., Oulad El Majdoub Y., Cacciola F., Mondello L., Aazza M., Zair T., Bouissane L. (2022). Phenolic composition, antioxidant and antibacterial activities of extract from flowers of *Rosa damascena* from Morocco. Separations.

[45] Ni M., Chen J., Fu M., Li H., Bu S., Hao X., Gu W. (2024). UPLC-ESI-MS/MS-based analysis of various edible Rosa fruits concerning secondary metabolites and evaluation of their antioxidant activities. Foods.

[46] Olennikov D.N., Chemposov V.V., Chirikova N.K. (2021). Metabolites of prickly rose: Chemodiversity and digestive-enzyme-inhibiting potential of Rosa acicularis and the main ellagitannin rugosin D. Plants.

[47] Guo Y., Zhao W., He Y., Li A., Feng Q., Tian L. (2024). Research on the pharmacognostic characteristics, physicochemical properties and in vitro antioxidant potency of Rosa laxa Retz. flos. Microsc. Res. Tech..

[48] Novikov O.O., Pisarev D.I., Zhilyakova E.T., Novikova M.U., Bondarenko E.V., Fadeeva D.A., Bezmenova M.D. (2011). Phytochemical study of the north caucasian Rosa spinossima L. fruit. Bull. Exp. Biol. Med..

[49] Özek G., Chidibayeva A., Ametov A., Nurmahanova A., Özek T. (2022). Chemical composition of flower volatiles and seeds fatty acids of Rosa iliensis Chrshan, an endemic species from Kazakhstan. Rec. Nat. Prod..

[50] White T.J., Bruns T., Lee S., Taylor J. (1990). Amplification and Direct Sequencing of Fungal Ribosomal RNA Genes for Phylogenetics. PCR Protocols: A Guide to Methods and Applications.

[51] Bolger A.M., Lohse M., Usadel B. (2014). Trimmomatic: A flexible trimmer for Illumina sequence data. Bioinformatics.

[52] Dierckxsens N., Mardulyn P., Smits G. (2017). NOVOPlasty: De novo assembly of organelle genomes from whole genome data. Nucleic Acids Res..

[53] Tillich M., Lehwark P., Pellizzer T., Ulbricht-Jones E.S., Fischer A., Bock R., Greiner S. (2017). GeSeq—Versatile and accurate annotation of organelle genomes. Nucleic Acids Res..

[54] Lohse M., Drechsel O., Bock R. (2007). OrganellarGenomeDRAW (OGDRAW): A tool for the easy generation of high-quality custom graphical maps of plastid and mitochondrial genomes. Curr. Genet..

[55] Beier S., Thiel T., Münch T., Scholz U., Mascher M. (2017). MISA-web: A web server for microsatellite prediction. Bioinformatics.

[56] Frazer K.A., Pachter L., Poliakov A., Rubin E.M., Dubchak I. (2004). VISTA: Computational tools for comparative genomics. Nucleic Acids Res..

[57] Amiryousefi A., Hyvönen J., Poczai P. (2018). IRscope: An online program to visualize the junction sites of chloroplast genomes. Bioinformatics.

[58] Rozas J., Sánchez-DelBarrio J.C., Messeguer X., Rozas R. (2003). DnaSP, DNA polymorphism analyses by the coalescent and other methods. Bioinformatics.

[59] Nguyen L.T., Schmidt H.A., Von Haeseler A., Minh B.Q. (2015). IQ-TREE: A fast and effective stochastic algorithm for estimating maximum-likelihood phylogenies. Mol. Biol. Evol..

[60] Nguyen H.Q., Nguyen T.N.L., Doan T.N., Nguyen T.T.N., Phạm M.H., Le T.L., Sy D.T., Chu H.H., Chu H.M. (2021). Complete chloroplast genome of novel Adrinandra megaphylla Hu species: Molecular structure, comparative and phylogenetic analysis. Sci. Rep..

[61] Oyuntsetseg D., Nyamgerel N., Baasanmunkh S., Oyuntsetseg B., Bayarmaa G.A., Choi H.J. (2024). The complete chloroplast genome of Swertia obtusa (Gentianaceae) in Mongolia. J. Asia Pac. Biodivers..

[62] Chen J.F., Wang S.Q., Cai H.W., Zhu Z.M. (2022). Characteristics and phylogenetic analysis of the complete chloroplast genome of Rosa glomerata (Rosaceae). Mitochondrial DNA B.

[63] Jian H., Zhang S., Zhang T., Qiu X., Yan H., Li S., Wang Q., Tang K. (2018). Characterization of the complete chloroplast genome of a critically endangered decaploid rose species, *Rosa praelucens* (Rosaceae). Conserv. Genet. Resour..

[64] Zhao X., Gao C. (2020). The complete chloroplast genome sequence of Rosa minutifolia. Mitochondrial DNA B.

[65] Gao C., Wu C., Zhang Q., Wu M., Chen R., Zhao Y., Guo A., Li Z. (2020). Sequence and phylogenetic analysis of the chloroplast genome for Rosa xanthina. Mitochondrial DNA B.

[66] Jian H.Y., Zhang Y.H., Yan H.J., Qiu X.Q., Wang Q.G., Li S.B., Wang Q.-G., Tang K.-X. (2018). The complete chloroplast genome of a key ancestor of modern roses, Rosa chinensis var. spontanea, and a comparison with congeneric species. Molecules.

[67] Yang T., Sahu S.K., Yang L., Liu Y., Mu W., Liu X., Strube M.L., Liu H., Zhong B. (2022). Comparative analyses of 3,654 plastid genomes unravel insights into evolutionary dynamics and phylogenetic discordance of green plants. Front. Plant Sci..

[68] Millen R.S., Olmstead R.G., Adams K.L., Palmer J.D., Lao N.T., Heggie L., Kavanagh T.A., Hibberd J.M., Gray J.C., Morden C.W. (2001). Many parallel losses of infA from chloroplast DNA during angiosperm evolution with multiple independent transfers to the nucleus. Plant Cell.

[69] Gao W., Zhou X., Yu Q., Lin G., Fu C., Kang T., Zeng H. (2024). Assembly and comparative analyses of the chloroplast genomes of the threatened plant Rosa anemoniflora. Forests.

[70] Jansen R.K., Saski C., Lee S.B., Hansen A.K., Daniell H. (2011). Complete plastid genome sequences of three rosids (Castanea, Prunus, Theobroma): Evidence for at least two independent transfers of rpl22 to the nucleus. Mol. Biol. Evol..

[71] Tangphatsornruang S., Uthaipaisanwong P., Sangsrakru D., Chanprasert J., Yoocha T., Jomchai N., Tragoonrung S. (2011). Characterization of the complete chloroplast genome of Hevea brasiliensis reveals genome rearrangement, RNA editing sites and phylogenetic relationships. Gene.

[72] Liu T.J., Zhang C.Y., Yan H.F., Zhang L., Ge X.J., Hao G. (2016). Complete plastid genome sequence of Primula sinensis (Primulaceae): Structure comparison, sequence variation and evidence for accD transfer to nucleus. PeerJ.

[73] Yang L., Deng S., Zhu Y., Da Q. (2023). Comparative chloroplast genomics of 34 species in subtribe Swertiinae (Gentianaceae) with implications for its phylogeny. BMC Plant Biol..

[74] Lei W., Ni D., Wang Y., Shao J., Wang X., Yang D., Wang J., Chen H., Liu C. (2016). Intraspecific and heteroplasmic variations, gene losses and inversions in the chloroplast genome of Astragalus membranaceus. Sci. Rep..

[75] Li C., Cai C., Tao Y., Sun Z., Jiang M., Chen L., Li J. (2021). Variation and evolution of the whole chloroplast genomes of Fragaria spp. (Rosaceae). Front. Plant Sci..

[76] Niu Z., Lin Z., Tong Y., Chen X., Deng Y. (2023). Complete plastid genome structure of 13 Asian Justicia (Acanthaceae) species: Comparative genomics and phylogenetic analyses. BMC Plant Biol..

[77] Asaf S., Khan A.L., Khan M.A., Shahzad R., Lubna, Kang S.M., Al-Harrasi A., Al-Rawahi A., Lee I.-J., Budak H. (2018). Complete chloroplast genome sequence and comparative analysis of loblolly pine (Pinus taeda L.) with related species. PLoS ONE.

[78] Nazareno A.G., Carlsen M., Lohmann L.G. (2015). Complete chloroplast genome of Tanaecium tetragonolobum: The first Bignoniaceae plastome. PLoS ONE.

[79] Li C., Zheng Y., Huang P. (2020). Molecular markers from the chloroplast genome of rose provide a complementary tool for variety discrimination and profiling. Sci. Rep..

[80] Hu L., Wang J., Wang X., Zhang D., Sun Y., Lu T., Shi W. (2024). Development of SSR markers and evaluation of genetic diversity of endangered plant Saussurea involucrata. Biomolecules.

[81] Yermagambetova M., Almerekova S., Turginov O., Sultangaziev O., Abugalieva S., Turuspekov Y. (2023). Genetic diversity and population structure of Juniperus seravschanica Kom. collected in Central Asia. Plants.

[82] Ye L., Shavvon R.S., Qi H., Wu H., Fan P., Shalizi M.N., Khurram S., Davletbek M., Turuspekov Y., Liu J. (2024). Population genetic insights into the conservation of common walnut (Juglans regia) in Central Asia. Plant Divers..

[83] Nagel J.C., Poletto T., Muniz M.F.B., Poletto I., de Oliveira J.N.M., Stefenon V.M. (2023). Species-specific plastid SSR markers reveal evidence of cultivar misassignments in Brazilian pecan [Carya illinoinensis (Wangenh.) K. Koch] orchards. Genet. Resour. Crop Evol..

[84] Götz J., Caré O., Beck W., Gailing O., Hosius B., Leinemann L. (2024). A novel set of chloroplast SSR markers for the genus Juglans reveals within species differentiation. Silvae Genet..

[85] Götz J., Leinemann L., Gailing O., Hardtke A., Caré O. (2024). Development of a highly polymorphic chloroplast SSR set in Abies grandis with transferability to other conifer species—A promising toolkit for gene flow investigations. Ecol. Evol..

[86] Liu H., Jacquemyn H., Wang Y., Hu Y., He X., Zhang Y., Zhang Y., Huang Y., Chen W. (2025). Comparative analysis of the chloroplast genomes of Cypripedium: Assessing the roles of SSRs and TRs in the non-coding regions of LSC in shaping chloroplast genome size. Int. J. Mol. Sci..

[87] Alharbi S.A., Albokhari E.J. (2025). Complete chloroplast genomes of Desmidorchis penicillata (Deflers) Plowes and Desmidorchis retrospiciens Ehrenb.: Comparative and phylogenetic analyses among subtribe Stapeliinae (Ceropegieae, Asclepiadoideae, Apocynaceae). Nord. J. Bot..

[88] Almerekova S., Yermagambetova M., Osmonali B., Vesselova P., Turuspekov Y., Abugalieva S. (2024). Complete plastid genome sequences of four Salsoleae s.l. species: Comparative and phylogenetic analyses. Biomolecules.

[89] Almerekova S., Shchegoleva N., Abugalieva S., Turuspekov Y. (2020). The molecular taxonomy of three endemic Central Asian species of Ranunculus (Ranunculaceae). PLoS ONE.

[90] Antil S., Abraham J.S., Sripoorna S., Maurya S., Dagar J., Makhija S., Bhagat P., Gupta R., Sood U., Lal R. (2023). DNA barcoding, an effective tool for species identification: A review. Mol. Biol. Rep..

[91] Jamdade R., Mosa K.A., El-Keblawy A., Al Shaer K., Al Harthi E., Al Sallani M., Al Jasmi M., Gairola S., Shabana H., Mahmoud T. (2022). DNA barcodes for accurate identification of selected medicinal plants (Caryophyllales): Toward barcoding flowering plants of the United Arab Emirates. Diversity.

[92] Cabelin V.L.D., Santor P.J.S., Alejandro G.J.D. (2015). Evaluation of DNA barcoding efficiency of cpDNA barcodes in selected Philippine Leea L. (Vitaceae). Acta Bot. Gall..

[93] Sevindik E., Korkom Y., Murathan Z.T. (2024). Evaluating DNA barcoding using cpDNA matK and rbcL for species identification and phylogenetic analysis of *Prunus armeniaca* L. (Rosaceae) genotypes. Genet. Resour. Crop Evol..

[94] Roy S., Tyagi A., Shukla V., Kumar A., Singh U.M., Chaudhary L.B., Datt B., Bag S.K., Singh P.K., Nair N.K. (2010). Universal plant DNA barcode loci may not work in complex groups: A case study with Indian Berberis species. PLoS ONE.

[95] Zhang S.Y., Yan H.F., Wei L., Liu T.J., Chen L., Hao G., Wu X., Zhang Q.L. (2024). Plastid genome and its phylogenetic implications of Asiatic Spiraea (Rosaceae). BMC Plant Biol..

[96] Tang C., Chen X., Deng Y., Geng L., Ma J., Wei X. (2022). Complete chloroplast genomes of Sorbus sensu stricto (Rosaceae): Comparative analyses and phylogenetic relationships. BMC Plant Biol..

[97] Lu Q., Tian Q., Gu W., Yang C.X., Wang D.J., Yi T.S. (2024). Comparative genomics on chloroplasts of *Rubus* (Rosaceae). Genomics.

[98] Wu S., Ueda Y., Nishihara S., Matsumoto S. (2001). Phylogenetic analysis of Japanese Rosa species using DNA sequences of nuclear ribosomal internal transcribed spacers (ITS). J. Hortic. Sci. Biotechnol..

[99] Tuong L.Q., Tam T.V., Dong D.V., Duong T.D., Cuc D.T.K., Thu P.T.L., Luong D.T., Tuyen V.T.M., Giang N.V., Tuan N.T. (2020). Identification of Vietnamese native rose species by using internal transcribed spacers (ITS) sequencing. Plant Cell Biotech. Mol. Biol..

[100] Lee S.Y., Xu K.W., Huang C.Y., Lee J.H., Liao W.B., Zhang Y.H. (2022). Molecular phylogenetic analyses based on the complete plastid genomes and nuclear sequences reveal Daphne (Thymelaeaceae) to be non-monophyletic as current circumscription. Plant Divers..

