# Supplementary material for: Characterization of the Four Rosa L. Species from Kazakhstan Based on Complete Plastomes and Nuclear Ribosomal Internal Transcribed Spacer (ITS) Sequences
Source: Genes (Basel). 2025 Jul 22;16(8):852. doi: 10.3390/genes16080852 (PMC12386023; doi:10.3390/genes16080852)
Supplement: Supplementary file 1 [file genes-16-00852-s001.zip › Table S1.pdf]

Table S1. Number of samples used in the phylogenetic analyses based on different data sets

Species name	NCBI accession number		
	**plastome**	**ycf1**	**ITS**
Rosa acicularis	MK714016	MK714016	MK847473
Rosa acicularis (in this study)	PV330080	PV330080	PV789381
Rosa anemoniflora	OR502860	OR502860	KM353019
Rosa beggeriana	NC 079961	NC 079961	MK989667
Rosa canina	MN661140	MN661140	MW309394
Rosa chinensis	MZ636543	MZ636543	FJ384671
Rosa cymosa	MT471268	MT471268	MW309398
Rosa davurica	MW381769	MW381769	FJ527710
Rosa fedtschenkoana	NC 061268	NC 061268	MH712606
Rosa filipes	MT062883	MT062883	KM353083
Rosa glomerata	OM519307	OM519307	KM353093
Rosa graciliflora	OQ992658	OQ992658	MW494600
Rosa iliensis (in this study)	PV330081	PV330081	PV789382
Rosa laevigata	MN372205	MN372205	FJ416663
Rosa lasiosepala	MK533318	MK533318	KM353123
Rosa laxa	na	na	PP906946
Rosa laxa (in this study)	PV330082	PV330082	PV789383
Rosa longicuspis	PP565789	PP565789	KM353131
Rosa lucidissima	MK782979	MK782979	KM353137
Rosa lucieae	MG727864	MG727864	KM353135
Rosa maximowicziana	MG727865	MG727865	MK847509
Rosa moyesii	OQ735285	OQ735285	KM353149
Rosa omeiensis	OL405263	OL405263	MW408864
Rosa roxburghii	KX768420	KX768420	MH711604
Rosa soulieana	OQ735268	OQ735268	FJ527716
Rosa spinosissima	na	na	KM353174
Rosa spinosissima (in this study)	PV330083	PV330083	PV789384
Rosa transmorrisonensis	MT681382	MT681382	KM353223
Dasiphora fruticosa	OQ992647	OQ992647	MZ366409
Fragaria pentaphylla	KY434061	KY434061	AM114833
